# Remote Programming of Adult and Pediatric Cochlear Implant Recipients: Clinical Trial Results

**DOI:** 10.1097/ONO.0000000000000073

**Published:** 2025-07-14

**Authors:** Sara Morton, Bob Dwyer, Noel Dwyer, Laura Holden, Krista Iannuzzi, Kristen Lewis, Morgan Nelson, Christine Brown, Smita Agrawal, Amy Stein, Carla Passmore, Jason Galster, Sarah Zlomke, Jill Firszt

**Affiliations:** 1 Austin Ear, Nose & Throat Clinic, Austin, TX; 2 Advanced Bionics, LLC, Valencia, CA; 3 Department of Otolaryngology, Washington University School of Medicine, St. Louis, MO; 4 Department of Otolaryngology, University of Colorado, Boulder, CO; 5 Midwest Ear Institute, Kansas City, MO; 6 Department of Otolaryngology, Vanderbilt University Medical Center, Nashville, TN.

**Keywords:** Clinical trial, Cochlear implant, Remote programming

## Abstract

**Objective:**

To evaluate the efficacy and safety of remote cochlear implant (CI) programming. The primary efficacy objective was to demonstrate that speech recognition in quiet after remote fitting is no worse than speech recognition in quiet after in-person office fitting. The primary safety endpoint was the absence of unanticipated adverse device effects related to remote programming.

**Study design and setting:**

Prospective within-subjects interventional study at 5 US centers.

**Participants:**

17 CI recipients (12 electric-only [EO] hearing; 5 with aidable residual hearing) with a minimum of 6 months of CI experience.

**Intervention:**

Programming conducted in person and remotely via a smartphone application.

**Main outcome measures:**

Speech recognition, fitting duration, and subjective questionnaires.

**Results:**

In the EO cohort, mean AzBio speech recognition for in-person created programs was 89.28% (SE = 3.48), compared with 91.94% (SE = 2.76) for programs created remotely. The combined EO and aidable residual hearing cohort’s mean speech recognition for programs created in person was 89.04% (SE = 2.66) versus 90.99% (SE = 2.09) for remotely created programs. The observed *P* value for EO and pooled cohorts was <0.001, indicating that speech recognition in quiet after remote fitting is no worse than that after in-person fitting. Absolute differences in fitting durations between programming methods ranged from 3 to 11 minutes. The total time spent for a typical CI office visit ranged from 1 to more than 4 hours for 88.3% of study participants.

**Conclusions:**

Remote programming provides noninferior outcomes to in-person programming and represents an important step toward improving the accessibility and affordability of obtaining audiology services by eliminating the need to travel. Participants and audiologists rated remote programming positively.

According to the 2021 World Health Organization’s *World Report on Hearing*, unaddressed hearing loss is the third leading cause of disability worldwide. More than 1.5 billion people currently experience some degree of hearing loss. An estimated US$1 trillion is lost annually due to the insufficient management of hearing loss. While this financial impact is significant, at the individual level, unaddressed hearing loss has substantial consequences on listening and communication, receptive and expressive language development, cognition, education, employment, social isolation/loneliness, and mental health that are difficult to measure (1). Despite these consequences, many individuals do not receive the interventions they need, such as cochlear implantation, which remains underutilized.

Cochlear implantation is considered best practice for individuals with unilateral severe, profound, or moderate sloping to profound sensorineural hearing loss (SNHL) (2); however, cochlear implant (CI) utilization rates remain low. Estimates of CI utilization rates in the United States for adults and children with severe-to-profound hearing loss range from 5.6% to 12.7% (3,4). In an international investigation of CI utilization rates in developed nations, Sorkin and Buchman (5) reported adult utilization rates of less than 10% in all 6 developed countries studied (ie, Australia, Austria, Germany, Sweden, the United Kingdom, and the United States). Pediatric CI utilization was much higher than that of adults in developed countries, but was considerably variable, ranging from 50% utilization in the United States to 98% in Australia. Differences among adult and pediatric utilization rates reflect the size of the potential CI candidate base, indicating a much larger number of adults who are appropriate CI candidates, but also the impact of timely referrals informed by newborn hearing screening programs and reimbursement programs available to young children.

Complexities in treatment delivery have also been acknowledged as part of the reason for the underutilization of CIs (3,6). About 3.4 billion people, or 43% of the world’s population, live in rural areas (7). In developed nations, such as the United States, about 60 million people (1 in 5 Americans) live in rural areas (8). Older residents are more likely to live in rural areas where healthcare is often less accessible (9,10). For example, over 80% of US veterans reside more than 180 miles from the nearest Veterans Health Administration facility providing CI services (11). These geographical challenges are significant in the context of CI services, as a profile of the catchment area of large US CI centers revealed an association between greater travel distances and older age at cochlear implantation (9).

Insufficient access to audiological services is even more pronounced in developing nations. In the World Health Organization African Region, 78% of countries have fewer than one audiologist per 1 million people. Similarly, in Southeast Asian nations, the density is less than 5 audiologists per million people, with 44% of these countries having fewer than one audiologist per million people (1).

Telehealth refers to using communications technologies to provide health care from a distance. Accessibility, convenience, efficiency, and reduced costs have been drivers in telehealth uptake (12,13). The COVID-19 pandemic accelerated the adoption of telehealth as the primary means of seeing noncritical patients, with healthcare providers quickly adapting to digital care (14). This shift has highlighted the benefits of telehealth, including increased access to care, enhanced patient care, convenience, reduced travel, and cost-effectiveness.

Telehealth in audiology has increasingly been employed in audiology clinics over the last 2 decades. Virtual audiology services, not limited to hearing aid fittings (15), newborn hearing screenings (16,17), interoperative monitoring (18), and CI mappings (19–21) are feasible and effective, with both audiologists and patients reporting high satisfaction (20,22,23).

As follows, for individuals who successfully navigate the cochlear implantation process despite the geographic and economic constraints, keeping up with the numerous postactivation programming and (re)habilitation appointments can be a significant hurdle. Extending audiology services via a remote programming solution to underserved rural and remote areas is one step toward improving the accessibility and affordability of obtaining audiology services. In this context, affordability can be extended to include not only reduced or lost wages and the possible need for additional childcare for patients traveling to in-person visits but also the financial burden of gas and wear and tear on vehicles, as well as the potential necessity for overnight hotel stays.

The first attempts at providing programming remotely involved placing programming equipment at a satellite facility. This has been demonstrated as safe and effective in adults (20,21,24) and children (19,25,26), but still requires recipients to travel to a designated remote site equipped with the necessary technology (eg, programming hardware, software, teleconference equipment) and a facilitator or other support staff. While reducing the distance compared with traveling to a central clinic, this still imposes significant travel requirements on patients, especially those living in very remote or rural areas. Alternatively, a laptop with the programming software and equipment could be mailed to the CI recipient ahead of a remote programming session to provide access to remote programming for individuals with logistical challenges traveling to the remote site. This approach offers several advantages, namely, eliminating the need for travel, which is more cost-effective, and flexibility for scheduling. However, it does not come without challenges, including some level of technical savviness to set up and use the equipment, consideration for returning the equipment, and potential loss or damage of expensive clinical hardware during shipping. Additionally, the costs associated with employing a facilitator or additional staff and the expenses related to shipping programming equipment may not be cost-effective and could pose a significant barrier to successfully implementing remote programming in clinic settings.

## STUDY AIM

The objective of the present study was to evaluate outcomes with a remote programming solution that would enable audiologists to wirelessly connect to a patient’s sound processor via a smartphone app to perform follow-up fittings in real time for unilateral or bilateral CI recipients. This programming solution could eliminate the need for CI recipients to travel to a satellite location for programming and the necessity to ship/return programming hardware. Specifically, the study aimed to determine whether speech recognition in quiet is noninferior when measured with programs created during a remote fitting session and an in-person office setting. We hypothesized that sentence recognition in quiet would be no worse with the program fitted remotely compared with the program fitted in person. In addition to speech recognition scores, psychophysical data, and fitting durations, information from subjective questionnaires was collected from both participants and investigators.

## MATERIALS AND METHODS

### Study Design

Beginning in July of 2021, a multicenter pivotal clinical trial to evaluate the safety and efficacy of CI remote fitting was conducted at 5 experienced centers in the United States. The Food and Drug Administration and the associated participating study center institutional review boards approved the investigational plan and informed consent form. All study procedures complied with ethical standards outlined in the Declaration of Helsinki. The study employed a prospective, within-participants, repeated-measures design to determine whether hearing performance is similar with programs created via a remote programming session and an in-person programming visit. The study used a noninferiority design to determine whether speech recognition in quiet with a program created in a remote session was no worse than speech recognition in quiet with a program created in an in-person setting. The primary efficacy endpoint was the comparison of AzBio sentence recognition in quiet after chronic use of a program fitted in person to chronic use of a program fitted with remote programming. Given previous work in this area, we hypothesized that sentence recognition with the investigational remote fitting program would be no worse than with the control in-person fitting program.

### Study Population

#### Inclusion Criteria

This study enrolled CI recipients with a HiResolution Bionic Ear System implant (Advanced Bionics, Valencia, CA). To participate in the study, recipients needed to be 13 years of age or older, have at least 6 months of CI use experience, a minimum of 1 month of experience with a Naída CI M or Sky CI M sound processor, moderate open-set speech recognition abilities with the implant alone (≥60% AzBio sentences in quiet), and score a minimum average of ≥3 on the Mobile Device Proficiency Questionnaire (MDPQ-16) (27).

#### Exclusion Criteria

The following exclusion criteria were used: 1) clinical presentation indicative of potential device malfunction; 2) unrealistic expectations regarding potential benefits, risks, and limitations of the investigational device as determined by the investigator; 3) unwillingness or inability of the participant to comply with all investigational requirements as determined by the investigator.

#### Cohort Assignment

Participants who met these criteria were enrolled in either the electric-only (EO) or the aidable residual hearing (ARH) groups based on the level of residual hearing in their implanted ear(s). Participants with residual low-frequency hearing sensitivity (pure-tone average [PTA] < 70 dB hearing level [HL] for 125, 250, and 500 Hz) and a severe-to-profound high-frequency SNHL (PTA of ≥ 70 dB HL for 1000, 2000, 3000, 4000, and 8000 Hz) in the implanted ear for unilaterally implanted participants and in both ears for bilaterally implanted participants were assigned to the ARH group. Bilaterally implanted recipients meeting the ARH criteria in only one ear were excluded. Recipients who did not meet the ARH criteria were fitted under the EO criteria.

### Study Intervention

In the control condition, participants were programmed with an investigational sound processor in the audiologist’s office (in-person) using a research version of the fitting software for in-person programming of the sound processors’ electric and combined electric and acoustic modes. This in-person fitting included measurement of impedances, measurement of electrode-specific neural response imaging, programming of upper stimulation levels (M level), and live speech stimulation to confirm the acceptability of adjustments. Tone burst stimulation mode was used to measure M levels on each active electrode. The following settings from each participant’s clinical (baseline) program were not modified: strategy, pulse width setting (eg, manual, APW II), filter type (eg, standard, extended low), radio frequency lock (eg, manual, AutoVoltage), and program options (eg, input dynamic range, ClearVoice, and SoftVoice settings). For participants in the ARH cohort, their current audiogram was entered into the fitting software at visit 1, and the fitting software calculated the acoustic gains. Starting just below or at the M level from the baseline program, each electrode was stimulated, with the stimulation level increased or decreased until the participant indicated the most comfortable loudness was reached.

The program was then stimulated in live speech mode to verify that the levels and settings were appropriate for everyday use. For ARH participants, this meant also confirming that the acoustic settings were adequately loud and comfortable. If required, adjustments were made to stimulation levels or options (eg, global or fine-tuned M levels, acoustic gains, acoustic and electric cutoff) to ensure the program was acceptable to each participant in terms of volume and sound quality.

**FIG. 1.**
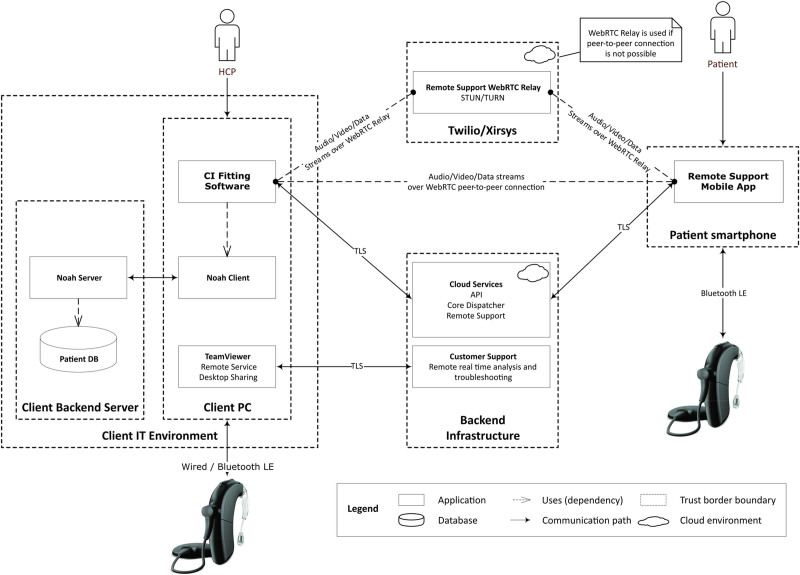
Remote fitting via a smartphone app. Data protection complies with the Health Insurance Portability and Accountability Act (HIPAA), General Data Protection Regulation (GDPR), and Swiss Federal Data Protection Act (FDRA) policies.

During the investigational condition (remote programming), the same investigator-initiated programming adjustments were transmitted over the internet to a cloud service, which forwarded the programming command to the research version of a mobile app through a Wi-Fi or cellular connection. The programming command was transmitted from the mobile app to the sound processor(s) via a standard Bluetooth Low Energy 2.4 GHz wireless connection, as illustrated in Figure 1.

#### Visit 1 Procedures (In-Person Fitting Session)

The study was conducted over 3 visits, as illustrated in Table 1. Following informed consent, demographics and hearing history were collected. All participants completed an MDPQ-16 and a custom reimbursement questionnaire. Standard audiometric testing was completed to determine cohort eligibility (ie, EO or ARH groups). Speech testing was conducted using the participant’s personal sound processor to confirm that the participant had at least moderate open-set speech recognition abilities with the implant alone (≥60% of words correct on the AzBio in quiet test).

**TABLE 1.** Schedule of study procedures

Procedure	Visit 1	Visit 2	Visit 3
Informed consent/assent	✓		
Demographics and audiological history	✓		
Mobile Device Proficiency Questionnaire (MDPQ-16)	✓		
Reimbursement Questionnaire	✓		
Audiometric thresholds	✓		
Speech in quiet, personal sound processor	✓		
Confirmation of eligibility	✓		
In-person office fitting	✓		
Remote programming via smartphone app		✓	
Acute AzBio in quiet	✓	✓	
Chronic AzBio in quiet		✓	✓
Subjective Outcome Measure Questionnaire (SSQ-12)		✓	✓
Participant Remote Fitting Satisfaction Questionnaire		✓	
Audiologist Remote Fitting Satisfaction Questionnaire		✓	
Adverse event assessment	✓	✓	✓

Participants who met the inclusion criteria were fitted with a study-dedicated sound processor in the audiologist’s office using a research version of the fitting software. This software is functionally similar to the commercially approved fitting software. ARH participants were fitted with an acoustic earhook and EO participants were fitted with a specialized microphone (referred to as a T-Mic) positioned at the entrance of the ear canal.

After the in-person fitting, acute speech recognition was tested in quiet to confirm the fitting of the investigational sound processor. Following the visit, participants tested the sound processor chronically for 2–3 weeks during their day-to-day activities.

#### Visit 2 Procedures (Remote Fitting Session)

Following the chronic take-home period, participants returned for visit 2, where they first completed an abbreviated version of the Speech, Spatial, and Qualities of Hearing questionnaire (SSQ-12) (28) to capture their listening experiences with the in-person program during the chronic period. Speech recognition was then evaluated in quiet with the same program. Next, a research version of the remote programming mobile app was installed on study-dedicated smartphones and was used to remotely program the electric and combined electric and acoustic modes of the sound processor(s) in the EO and ARH cohorts, respectively. Participants were placed in a remote location within each site’s clinical area and communicated with the audiologist through the mobile app on a study-dedicated smartphone. The audiologist communicated with the participant via a research version of the fitting software. When programming was completed, acute speech recognition testing in quiet was performed in a sound booth to confirm the efficacy of the fitting.

The audiologists and participants completed a custom remote fitting satisfaction questionnaire at the end of the visit. Following this remote programming session, participants tested the remotely programmed sound processor chronically for 2–3 weeks during their day-to-day activities.

#### Visit 3 Procedures

Visit 3 occurred 2–3 weeks after visit 2. At the beginning of the visit, participants completed the SSQ-12 questionnaire to capture their listening experiences with the remotely programmed sound processor during the chronic period. Speech recognition testing with this program was completed in quiet. Participants then returned the study-dedicated sound processor and returned to using their baseline clinical sound processor.

### Study Eligibility

#### Mobile Device Proficiency Questionnaire Questionnaire

The MDPQ-16 was used to determine the mobile device proficiency of the participants who sought to participate. This questionnaire asks about the ability to perform several tasks with a mobile device. The MDPQ-16 includes 16 items classified into 8 subscales: mobile device basics, communication, data and file storage, internet, calendar, entertainment, privacy, troubleshooting, and software management. Each subscale contains 2 questions for which the answer could be “1—never tried,” “2—not at all,” “3—not very easily,” “4—somewhat easily,” and “5—very easily.”

### Primary Outcome Measure

#### Sentence Recognition

Speech recognition testing in quiet was completed with 2 lists of AzBio sentence materials (29) presented at 65 dBA at the participant’s location from a single loudspeaker at 0° azimuth. Testing was performed unilaterally for unilaterally implanted participants and bilaterally for bilaterally implanted participants. Contralateral ear devices were removed for unilaterally implanted participants, and foam plugs were used to isolate the test ear, if necessary. Scores were the average of the percent correct scores from the 2 lists.

### Secondary Outcome Measures

#### Psychophysical and Electrophysiological Data

Psychophysical and electrophysiological measurements and fitting adjustments were logged during the fitting sessions to demonstrate the completion of the programming commands made in the programming software. Psychophysical data were collected individually at each fitting session on each implant side (right or left). Data included adjustments to the acoustic component for subjects in the ARH cohort, threshold neural response imaging (tNRI) measurements on each of a possible 4 electrodes (electrodes 3, 7, 11, 14), impedance measurements on each of a possible 16 electrodes (numbered 1–16), and confirmation of M Level (CU) changes.

#### Participant and Investigator Satisfaction Questionnaires

After the remote fitting session, a custom remote fitting satisfaction questionnaire was completed by both the participant and the audiologist. The participant questionnaire (for specifics, see Participant Remote Fitting Satisfaction Questionnaire, http://links.lww.com/ONO/A37) consisted of 10 multiple-choice questions divided into 3 subscales concerning participants’ satisfaction with the remote fitting experience: communication quality ratings, technology quality ratings, and overall rating. The responses could be “not applicable,” “1—strongly disagree,” “2—slightly disagree,” “3—neither agree nor disagree,” “4—slightly agree,” and “5—strongly agree.”

The investigator’s custom questionnaire (for specifics, see Investigator Remote Fitting Satisfaction Questionnaire, http://links.lww.com/ONO/A38) was completed by the programming audiologist and collected information regarding their satisfaction with the remote fitting experience. It consisted of 11 multiple-choice questions divided into 3 subscales: communication quality ratings, technology quality ratings, and overall ratings. The answers could be “not applicable,” “1—strongly disagree,” “2—slightly disagree,” “3—neither agree nor disagree,” “4—slightly agree,” and “5—strongly agree.”

#### Speech, Spatial, and Qualities of Hearing (SSQ-12)

Participants completed the SSQ-12, which was used to evaluate the chronic experience with programs created using each programming methodology in the participants’ everyday hearing environments. The SSQ is designed and validated for use typically as a complement to behavioral or experimental measures of hearing ability, and it includes 12 items regarding aspects of the ability and experience of hearing and listening in different situations. For each question, a rating from 0 to 10 is assessed, for which a rating of 10 means that the participant would be perfectly able to do or experience what is described in the question. In contrast, a rating of 0 means that the participant would be unable to do or experience what is described.

#### Reimbursement Questionnaire

Participants completed a custom reimbursement questionnaire consisting of 4 multiple-choice questions. Information was collected regarding transit time and distance from home to their CI center, total time spent during a typical CI office visit, and whether expenses are covered by insurance.

#### Additional Data Collection

In-person fitting and remote fitting durations were collected during the fitting sessions.

### Safety Management

Adverse event (AE) data were collected and evaluated during the study to ensure that participants’ safety was maintained as per the requirements of an investigational device exemption and the respective center’s institutional review board guidelines for reporting.

### Sample Size Determination

The required sample size was based on a power analysis using data from prior clinical trials showing a standard deviation of 6.24% for the primary efficacy endpoint. With a sample size of 8 complete pairs, there is 97% power to rule out a −10% null hypothesis supporting noninferiority for a 1-sided 2.5% test with statistical significance. This sample size calculation provided the minimum sample size for the EO cohort. An additional 5 ARH participants were enrolled in the study to provide clinical data for the electric and acoustic fitting mode for participants wearing an acoustic earhook. The total sample size was expanded to 17 participants (12 EO and 5 ARH) as the expected dropout rate due to withdrawals or loss to follow-up was not expected to exceed 4 participants.

### Statistical Analyses

Noninferiority analyses were based on paired *t* tests that looked at the differences in speech scores calculated using the scores obtained via the remotely created program minus the scores obtained from the in-person created program. A noninferiority bound of 10% was selected for the AzBio sentence test based on procedures used in previous clinical trials (30,31). Additionally, during validation of the AzBio sentence test, Spahr et al (29) showed that the 95% confidence intervals for average speech recognition for 2-list administration approximated 10%. Statistically significant test results that reject the null hypotheses, indicating that the means of the paired differences are greater than −10 percentage points for AzBio in quiet, will support the observation that remote programming is noninferior to in-person programming.

## RESULTS

### Participants

Nineteen study participants were enrolled across 5 study sites. All participants provided assent or informed consent. Two participants were excluded from participation. One failed for not meeting the inclusion criteria for speech recognition and the other for having an average score of <3 on the MDPQ-16, which fell below the qualification threshold of ≥3. Of the remaining 17 participants, 12 were enrolled in the EO group and 5 in the ARH group. Seven participants were implanted bilaterally. All 7 bilateral subjects were assigned to the EO group. Three participants were under the age of 18 years at the time of study enrollment. All 17 participants allocated to ARH and EO cohorts completed the study; there were no withdrawals or losses to follow-up. Additional group characteristics can be found in Table 2.

**TABLE 2.** Participant demographics for electric-only (EO) and aidable residual hearing (ARH) cohorts

	EO cohort	ARH cohort
Total participants	12	5
Age, y		
Mean (SD)	45.2 (22.9)	67.0 (7.2)
Median	49.5	68
Range	15–77^*a*^	60–78
Gender		
Male	5 (41.7%)	1 (20%)
Female	7 (58.3%)	4 (80%)
Implanted devices		
HiRes Ultra 3D	6 (25.0%)	1 (4.2%)
HiRes Ultra	1 (4.2%)	0 (0.0%)
HiRes 90K Adv.	3 (12.5%)	4 (16.7 %)
HiRes 90K	9 (37.5%)	0 (0.0%)

Implanted device percentages are based on the total number of implanted devices in the study (N = 24).

^*a*^Three participants were under the age of 18.

SD indicates standard deviation.

### Protocol Deviations and Adverse Events

Fourteen minor protocol deviations occurred with 10 participants among all 5 sites. These deviations were categorized as 1 “out-of-window visit,” 5 “test conditions not conducted per protocol,” 3 “informed consents performed incorrectly,” and 5 “other.” An example of a protocol deviation in the “other” category occurred when a participant’s personal processor information was loaded into the study software before obtaining informed consent. None of the deviations affected the efficacy and safety of the investigational device, software, or application.

During the study, 2 AEs were reported involving participants in the EO group. Neither of these AEs were deemed related to the study device or procedure. One participant, who had a history of chronic migraines triggered by weather changes, had to cancel and reschedule their second study visit due to a migraine, which subsequently resolved. Additionally, another participant was identified as potentially experiencing a device failure.

### Mobile Device Proficiency Questionnaire (MDPQ-16)

The MDPQ-16 was administered as part of the qualification procedure. The median MDPQ-16 score was 4.70, ranging from 3.1 (not very easily) to 5.0 (very easily), representing a wide range of mobile device experiences.

### Primary Outcome

#### Speech Recognition in Quiet

**FIG. 2.**
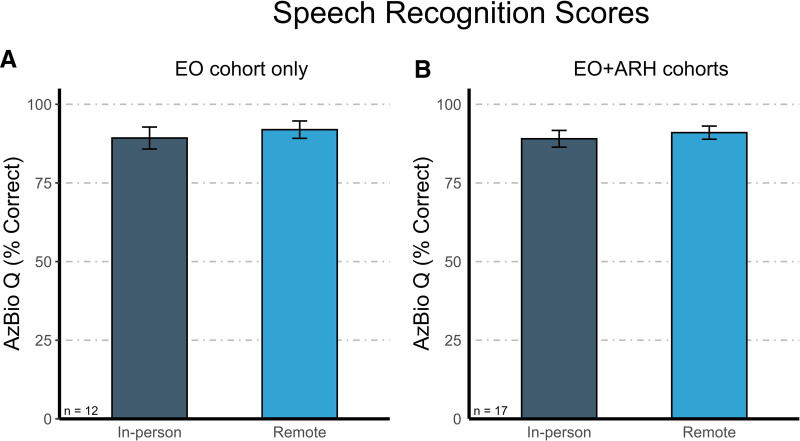
Speech recognition scores (in percent correct) for programs created using in-person (dark gray) and remote (blue) fitting methods. Error bars show the standard error of the mean. *A,* Results from the electric-only (EO) cohort (n = 12; primary efficacy endpoint). *B*, Results from the EO and aidable residual hearing (ARH) cohorts pooled (n = 17).

Figure 2A shows the mean AzBio sentence recognition scores in quiet after chronic use of the remotely fitted program and the in-person fitted program for the EO cohort (n = 12). The mean AzBio speech recognition score for the program created in person was 89.28% (SE = 3.48). The mean AzBio speech recognition for the program created remotely was 91.94% (SE = 2.76). This paired difference is 2.66 (SE = 0.99), with a 2-sided 95% confidence interval (0.48–4.84). The observed *P* value was <0.001, rejecting the null hypothesis of inferiority, confirming that sentence recognition with the remote programming solution is not significantly inferior to that with in-person fitting.

A noninferiority evaluation was also completed with the EO and ARH cohorts pooled together (N = 17; Fig. 2B). The mean speech recognition score for the program created in person was 89.04% (SE = 2.66), while the mean speech recognition score for the program created remotely was 90.99% (SE = 2.09). This paired difference equals 1.95 (SE = 1.31), with a 2-sided 95% confidence interval (−0.81 to 4.72). The observed *P* value was <0.001, thereby rejecting the null hypothesis of inferiority, again supporting that sentence recognition with remote programming solution is noninferior to in-person fittings.

#### Analyses of Individual Results

An analysis of the efficacy of remote fitting relative to in-person fitting based on individual data was completed. A binomial distribution model for the AzBio sentence test (29) was used to determine whether individual participants demonstrated a significant difference in speech recognition between the remotely fit and in-person fit programs. The expected variability was based on the test–retest variance established by Spahr et al (29). Any points that fell outside the established 95% confidence interval would be considered different.

Scores from 15 of the 17 participants (88%) fell within the 95% confidence interval, indicating no significant individual differences in speech recognition performance between the programming methodologies. Two participants had scores that fell outside the 95% confidence interval, indicating a difference in speech recognition performance between the 2 programs. Both participants achieved higher speech recognition outcomes with the in-person fit program. Mean performance on AzBio sentence materials for 1 participant in the ARH cohort was 93.5% for the in-person fit program and 80.8% for the remotely fitted program. Mean performance for a second participant in the EO cohort was 100% for the in-person fit program, compared with 97.5% for the remotely fit program. However, Spahr et al (29) urge caution in interpreting differences between scores when the reference score falls below 15% or above 85% due to compression from floor and ceiling effects. In the case of the EO participant with a reference score of 100% correct, anything less than 100% falls outside the 95% confidence interval for AzBio sentences.

### Secondary Outcomes

#### Psychophysical and Electrophysiological Data

**FIG. 3.**
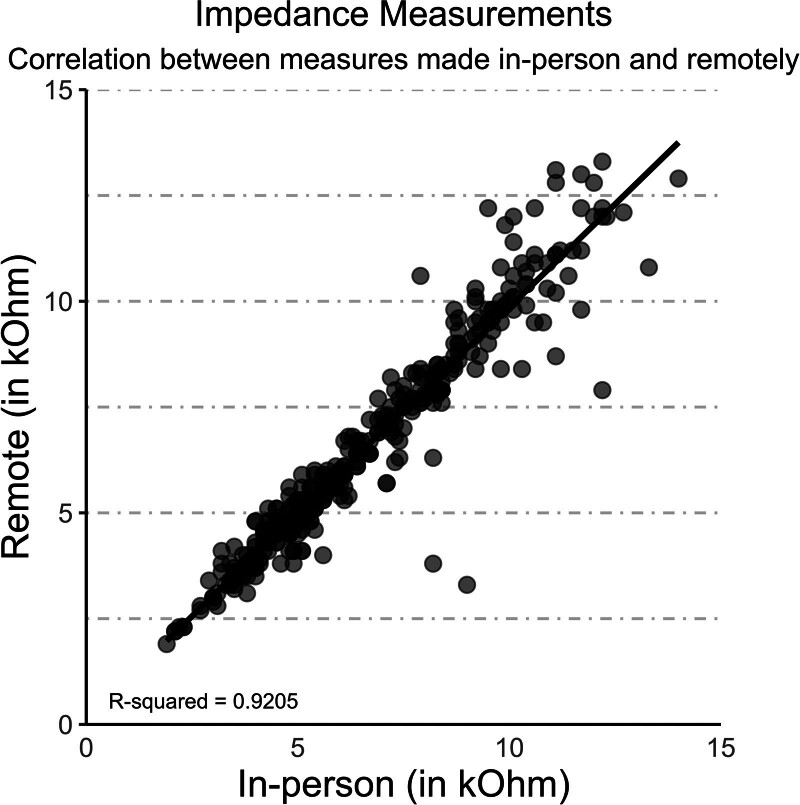
Comparison of impedance measurements obtained during the in-person and remote fittings. Each point represents a pair of impedance measurements, with the *x*-axis being the in-person measurement (in kOhm) and the *y*-axis being the remote measurement (in kOhm). The line represents the linear model fit to the data, indicating the relationship between in-person and remote measurements.

Psychophysical and electrophysiological measurements and fitting adjustments were logged during the fitting sessions and demonstrated that the majority of the commands were completed. Instances in which a command was not completed were exclusively related to a routine clinical consideration, such as a disabled electrode or a tNRI estimated threshold that fell outside the test level range included in the study protocol. The tNRI measurement could be conducted using equivalent clinical routines remotely and in-person. Figure 3 shows a record of impedance measurements from all participants.

#### Subjective Outcome Measures Questionnaire (SSQ-12)

**FIG. 4.**
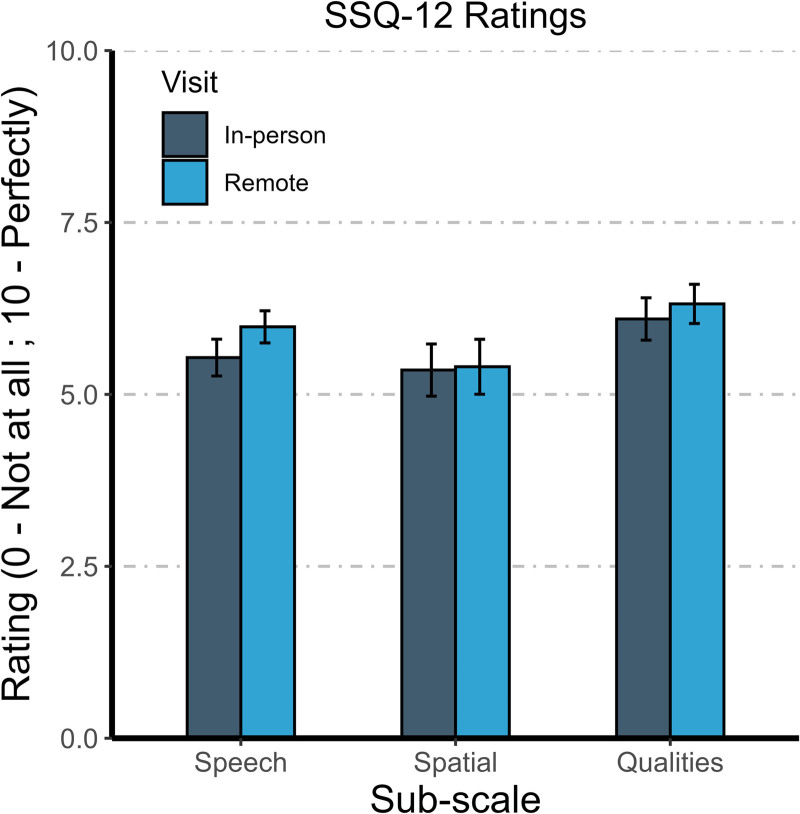
Mean results from the SSQ-12 completed at visits 2 and 3 for all participants pooled. Error bars show the standard error of the mean.

Figure 4 summarizes SSQ completed at visits 2 and 3 for all participants pooled. Between visits 2 and 3, the total SSQ score (not shown) across subscales increased for all cohorts. Specifically, mean scores for the EO cohort increased from 5.96 to 6.32, scores for the ARH cohort increased from 4.99 to 5.05, and scores for all participants pooled increased from 5.68 to 5.95.

#### Fitting Duration

For the study, investigators were asked to begin timing the fitting session when they clicked “Connect” in the fitting software to connect to the sound processor(s), and the session timing stopped when they clicked “Save & Close.” Unilateral CI participants were fitted remotely unilaterally, and bilateral CI recipients were fitted remotely bilaterally. Differences in mean overall fitting durations between in-person and remote programming fittings ranged from 3 to 11 minutes (Table 3).

**TABLE 3.** Fitting durations expressed in minutes

	Unilateral		Bilateral
	EO cohort	ARH cohort	EO cohort
Visit 1 (in-person)			
n	5	5	7
Mean (SD)	22.2 (6.0)	35.4 (22.98)	42 (20.88)
Median	19.2	24	46.8
Range	16.2–31.2	19.2–73.8	12.0–73.8
Visit 2 (remote)			
n	5	5	7
Mean (SD)	25.2 (7.32)	46.8 (35.1)	34.8 (14.1)
Median	27	40.8	31.2
Range	16.8–34.8	15.0–103.2	19.2–64.2

ARH indicates aidable residual hearing; EO, electric-only; SD, standard deviation.

#### Participant Reimbursement Questionnaire

When asked about the “Approximate time spent in total during a typical CI office visit” (eg, total spent in travel, wait, and appointment times), responses from the 17 participants ranged from less than 1 hour (11.8%), 1–2 hours (47.1%), 2–4 hours (29.4%), and more than 4 hours (11.8%). This distribution of responses indicated that the total time spent during a typical CI office visit ranged from 1 to more than 4 hours for 88.2% of study participants. When asked about the “Total distance I travel between home and my CI center (round trip mileage) is,” responses from the 17 participants ranged from less than 25 miles (35.3%), 26–50 miles (35.3%), 51–100 miles (23.5%), and more than 100 miles (5.9%). This response distribution indicates that travel distance to the clinic ranges from 26 miles to more than 100 miles for 64.7% of study participants. When asked if office visits related to CI care were covered by insurance, 41.2% of study participants were not reimbursed for all costs associated with a CI clinic visit. For example, the additional costs patients incur to physically travel to the center—like transportation, parking, or other incidental expenses—may not be covered by insurance.

### Participant and Audiologist Satisfaction Questionnaires

**FIG. 5.**
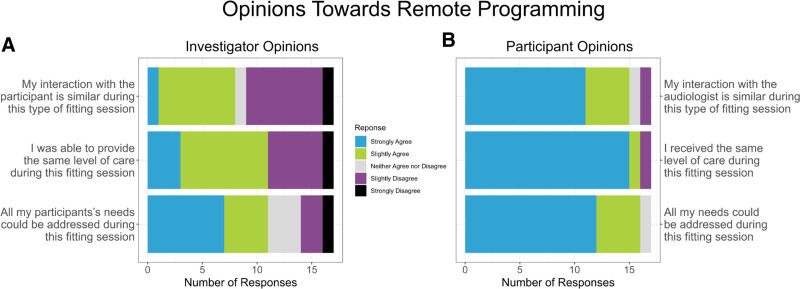
Audiologist (*A*) and participant (*B*) self-reported opinions towards remote programming.

Information regarding the investigator’s (Fig. 5A) and participant’s satisfaction with the remote programming experience (Fig. 5B) collected via custom questionnaires is shown in Figure 5. The results of the questionnaires, which involved rating the strength of agreement with specific statements, revealed that participant and audiologist ratings did show differences. For example, 16 of 17 participants agreed that “All my needs could be addressed during this fitting session,” indicating that most felt the remote fitting addressed their needs. However, fewer responses from audiologists (11/17) felt all their participants’ needs could be addressed during the fitting session. In response to the statement, “I received the same level of care during this fitting session,” nearly all participants (16/17) agreed, indicating that most felt that the remote fitting experience provided a similar level of care to an in-person clinic visit. Again, fewer responses from audiologists (11/17) felt that they could provide the same level of care during the remote fitting session. When asked to rate how much they agreed with the statement, “My interaction with the audiologist is similar during this type of fitting session,” 15 of 17 participants agreed, indicating that most study participants felt that the remote fitting experience was like an in-clinic fitting; however, responses among the investigators showed a wider range of opinions than the preceding questions. Slightly less than half of the responses from the investigators (8/17) agreed that their interaction with the participant was similar during a remote session.

## DISCUSSION

The result of this study shows that remote programming is a viable alternative to traditional in-person programming and offers an opportunity for time and cost savings, especially in situations where access to in-person care is challenging or limited. The primary efficacy objective was to demonstrate that speech recognition in quiet after remote fitting is no worse than speech recognition in quiet after in-person fitting. Data from the EO and EO-ARH cohorts pooled together show that the study meets the primary efficacy objective and noninferiority endpoint.

In the current study, approximately 94.1% of participants lived within 50 miles (one-way travel distance) of the participating research center. In the study by Nassiri et al (9) on the catchment area and patient profile of US-based CI centers, the reported median 1-way travel distance for patients who underwent CI surgery was 52 miles, suggesting that our sample may underestimate the distances traveled by the wider CI recipient population. Regardless, in both studies, a substantial proportion of CI recipients traveled significant distances for their CI care, highlighting the importance of remote programming in overcoming geographical challenges in delivering CI care. Additionally, it is important to recognize that the time it takes to travel to a programming center is not always directly related to how far someone lives from it. For instance, someone living in a busy urban area may live only a few miles from their clinic, but the travel time can be significant due to traffic, congestion, stoplights along their route, or parking. Thus, remote programming is expected to offer valuable time savings to recipients living in cities or suburban areas where traffic and travel delays are common.

During the study, 2 AEs were reported that were unrelated to the investigational software or remote programming. Device observations noted during the remote fitting study visit were related to the speed of the prototypical software interface or the bandwidth of the internet connection. All device observations were addressed and mitigated at the time of report or observation, except for 2 device observations resolved in a subsequent study visit. No unanticipated adverse device events were observed. A successful primary efficacy evaluation and the absence of device-related AEs indicate that remote fitting from AB is safe and effective.

One of the key findings of this study is that remote programming effectively addresses the needs of participants. Most participants reported that their needs could be adequately addressed during remote fitting sessions, highlighting the potential of remote programming to provide comprehensive care like their current in-person programming visits. This is particularly important in areas where access to audiological services is limited, as remote programming can help bridge the gap and provide essential care to individuals who might otherwise not have access to it.

Most responses from audiologists indicated that they achieved outcomes similar to those of in-person sessions. This suggests that remote programming can be an effective tool for audiologists in delivering care to their patients. In some instances, the investigators’ responses indicated an opinion that remote fitting would not be considered for all patient types and visits, suggesting that there may be limitations to the effectiveness of remote programming in some instances and that remote programming may not be a 1:1 substitute for in-person care, highlighting the importance of considering individual patient needs and the complexity of treatment when determining the suitability of remote programming versus in-person care. Future research could investigate the specific patient populations and situations where remote programming is the most beneficial. For example, examining patient-specific factors such as cognitive status, duration of deafness, age, dexterity, visual acuity, and speech recognition scores could help determine for whom remote programming could be a substitute for in-person clinical care. Additionally, research to determine when remote programming is most appropriate in the hearing rehabilitation process could help audiologists develop guidelines to ensure that patient care remains personalized and effective.

## CONCLUSION

Our study demonstrates that remote programming can be an effective tool for improving access to care and addressing complexities in treatment delivery for individuals with CIs. Remote programming utilizes synchronous telemedicine rendered through real-time interactive audio and video telecommunication, meeting Medicare’s current reimbursement requirements for telehealth at the time of this submission. However, access to reimbursement for remote programming can vary based on federal and state laws and regulations and payer policies. Additionally, more research is needed to explore the long-term effects of remote programming and to identify ways to optimize its use in clinical practice. Despite this, the findings from this clinical study show that remote programming is an effective innovation that provides recipients and audiologists with measurable benefits.

Specifically, the results demonstrate that:

- Speech recognition in quiet with remotely created programs is noninferior to speech recognition in quiet with programs created during an in-person fitting. This finding was observed for unilateral and bilateral, EO CI recipients and those with acoustic residual hearing in the implanted ear.
- Overall fitting durations were similar between in-person and remote fittings. For both in-person and remote fittings, the total fitting durations for bilateral and ARH fittings were longer than the respective unilateral and EO fittings, which is explained by additional time spent fitting the second ear or the acoustic ear hook component.
- Remote programming offers CI recipients time and cost savings compared with in-person routine clinic visits. When considering total time spent in travel, wait, and appointment times, the total time spent during a typical CI office visit ranged from 1 to more than 4 hours for 88.2% of study participants. Forty percent of participants in this study are not reimbursed for expenses related to a typical clinic visit.
- Research participants and audiologists rate remote programming positively. Participant and audiologist ratings did show differences. Specifically, participant ratings were higher on average, suggesting the remote fitting was like their in-person experience.
- Subjectively reported outcomes further support the efficacy analyses.

## ACKNOWLEDGMENT

This work was conducted while Dr. Sara Morton was affiliated with Austin Ear, Nose & Throat Clinic (Austin, TX). The authors would like to thank the participants for their time and commitment, as well as other key study personnel for their essential support in making this investigation successful.

## FUNDING SOURCES

This work was supported by a research grant from Advanced Bionics, LLC.

## CONFLICT OF INTEREST STATEMENT

J.F. is on the audiology advisory board for Advanced Bionics. Advanced Bionics employed B.D., S.A., A.S., C.P., and J.G. during this clinical investigation. The remaining authors declare no conflicts of interest.

## DATA AVAILABILITY STATEMENT

Data can be found online at https://clinicaltrials.gov/study/NCT05034731.

## Supplementary Material


