# Supplementary material for: Remote Programming of Adult and Pediatric Cochlear Implant Recipients: Clinical Trial Results
Source: Otol Neurotol Open. 2025 Jul 14;5(3):e073. doi: 10.1097/ONO.0000000000000073 (PMC12466901; doi:10.1097/ONO.0000000000000073)
Supplement: Supplementary file 2 [file ono-5-e073-s002.pdf]

Investigator Remote Fitting Questionnaire

Based on your experience during this fitting session, rate the quality of the communication between you and the subject for each condition below on a scale from 1-5, with 1 being "Strongly disagree," 3 being "Neither agree nor disagree" and 5 being "Strongly agree."

a. Communication between the parties was acceptable during this session

Based on your experience during this fitting session, for the following situations, rate the quality of the technology used during this fitting session for each condition below on a scale from 1-5, with 1 being "Strongly disagree," 3 being "Neither agree nor disagree" and 5 being "Strongly agree." If you did not interact with technology in the condition listed below, please select "N/A".

a. The technology was easy to use during this fitting session
b. I was able to complete this fitting session without additional technical assistance
c. I was able to overcome any technical issues encountered during the session (e.g., signal delays, internet speeds, connection problems).

Based on your experience during the fitting session, rate your strength of agreement with the statements below on a scale from 1-5, with 1 being "Strongly disagree," 3 being "Neither agree nor disagree" and 5 being "Strongly agree."

a. This type of fitting is acceptable
b. All my subject's needs could be addressed during this fitting session
c. I was able to provide the same level of care during this fitting session
d. My interaction with the subject is similar during this type of fitting session
e. I would want to use this type of fitting in the future on a regular basis
f. I would only want to use this type of fitting in the future if other options are impractical
g. I would recommend this type of fitting to my patients who use cochlear implants
h. Please explain how this fitting session did or did not meet your needs:
